# Nystatin Effectiveness in Oral Candidiasis Treatment: A Systematic Review & Meta-Analysis of Clinical Trials

**DOI:** 10.3390/life12111677

**Published:** 2022-10-22

**Authors:** Anamika Rai, Satya Ranjan Misra, Saurav Panda, Grzegorz Sokolowski, Lora Mishra, Rupsa Das, Barbara Lapinska

**Affiliations:** 1 Department of Oral Medicine & Radiology, Institute of Dental Sciences, Siksha ‘O’ Anusandhan University, Bhubaneswar 751003, India; 2 Department of Periodontics, Institute of Dental Sciences, Siksha ‘O’ Anusandhan University, Bhubaneswar 751003, India; 3 Department of Prosthetics, Medical University of Lodz, 251 Pomorska St., 92-213 Lodz, Poland; 4 Department of Conservative Dentistry & Endodontics, Institute of Dental Sciences, Siksha ‘O’ Anusandhan University, Bhubaneswar 751003, India; 5 Department of General Dentistry, Medical University of Lodz, 251 Pomorska St, 92-213 Lodz, Poland

**Keywords:** nystatin, oral candidiasis, denture stomatitis, systematic review, meta-analysis, photodynamic therapy, treatment duration

## Abstract

Oral candidiasis is the most common opportunistic fungal infection caused by commensal *Candida* species. Since there are various local and systemic predisposing factors for the disease, the treatment also varies from topical to systemic antifungal agents. Nystatin is a common antifungal agent used topically. The aim of this systematic review was to evaluate and compare the efficacy of different antifungal agents and the safety of nystatin in the treatment of oral candidiasis. Three electronic databases were searched for randomized controlled trials comparing nystatin with other anti-fungal therapies or placebo. Clinical and/or mycological cure was the outcome evaluation. A meta-analysis and descriptive study on the efficacy, treatment protocols, and safety of nystatin was also conducted. The meta-analysis included five studies, which compared the efficacy of nystatin suspensions with photodynamic therapy. A significant difference in the colony-forming units per milliliters (CFU/mL) of *Candida* species was observed at 60 days intervals for both palatal mucosa and denture surfaces, with both groups favoring nystatin with low heterogeneity at a 95% confidence interval. Nystatin and photodynamic therapy were found to be equally effective for the clinical remission of denture stomatitis as well as a significant reduction of CFU/mL of *Candida* species from dentures and palatal surfaces of the patients.

## 1. Introduction

The normal oral microflora is a complex population of diverse microorganisms consisting of eubacteria, mycoplasmas, fungi, archaea, and protozoa [1,2]. Fungi are free-living, eukaryotic organisms that may be in round, filamentous, or dimorphic forms, amongst which the *Candida* species are most frequently encountered by dentists [2,3]. The opportunistic infections in humans afflicted by *Candida albicans* and other related species can clinically manifest ranging from the most common oral thrush to fatal, systemic superinfections in patients with local or systemic predisposing factors [4,5,6]. Typically, the infection due to *Candida* is opportunistic, occurring due to various factors, including compromised host defense, or a break in the normal oral mucosa. In addition to that, other external factors like the use of broad-spectrum antibiotics or poor oral hygiene especially in denture wearers increase the probability of *Candida* infection [2,7]. Use of dentures favors oral infections due to continuous local micro-trauma and increased time of contact with micro-organisms. Both partial and complete denture wearers harbor micro-organisms especially *Candida* species [2,8]. The use of soft lining materials to increase the comfort and the fit of removable dentures are penetrated by the fungal hyphae, predisposing to *Candida* infection [6,9]. *Candida albicans* is the most frequently encountered *Candida* species that reports for more than 90% of the isolates from the oral cavity [4,10,11]. Other species that have been identified to be pathogenic to humans are *Candida parapsilosis*, *Candida tropicalis*, *Candida glabrata*, *Candida krusei*, *Candida guilliermondii*, and *Candida lusitaniae*. It has been reported that *Candid*a species are present in 26 to 75% of the healthy inhabitants in absence of any lesion as commensals in the oral cavity [4,12].

The management of oral candidiasis involves topical as well as systemic antifungal agents. For patients who are intolerant to or fail to respond to topical forms of treatment and are at higher peril of developing systemic infections, systemic antifungal agents like fluconazole, itraconazole, and miconazole are appropriate [13,14,15]. Because of various drug interactions as well as the reduced vulnerability of *Candida* species, except for *Candida albicans* towards azoles, the use of systemic antifungal agents is limited [14,15,16]. Therefore, for noninvasive cases of oral candidiasis, the first line of treatment is topical antifungal agents like nystatin, miconazole, amphotericin B, and clotrimazole [17,18]. Nystatin is produced by *Streptomyces noursei* strains, which is a membrane-active polyene macrolide. It is the most frequently prescribed topical antifungal drug by dentists and is available in various forms like topical creams, oral pastilles, and oral suspensions [17,18,19,20]. The drug is not absorbed from the gastrointestinal tract when orally administered eliminating the adverse effects [21]. Nystatin is usually administered in doses of 200,000–400,000 IU four times a day and 100,000–200,000 IU for infants and neonates for about 4 weeks, but there is no universally accepted dosage, formulation or duration of treatment for oral candidiasis [22,23,24,25,26]. Therefore, this study aims to summarize as well as evaluate the efficiency of different protocols of treatment (like dosage, formulations, and durations), along with the safety of nystatin in different inhabitants with oral candidiasis with the help of systematic review and meta-analysis.

## 2. Methods

The systematic review was performed under the Preferred Reporting Items for Systematic Reviews and Meta-Analyses guidelines [27]. This systematic review was registered on PROSPERO as CRD42021290307, and the search was completed by October 2021.

### 2.1. Inclusion Criteria

This review was restricted to randomized controlled trials in English language comparing nystatin in all topical forms to all other antifungal therapies or placebo. The clinical diagnosis was the basis of diagnosing oral candidiasis with or without mycological test confirmation. Primary outcomes were the clinical response rate defined as the cure or improvement of signs and symptoms attributable to the oral lesion as well as mycological cure rate defined as the negative culture’s result. Secondary outcomes were the relapse rate, the incidence of systemic infections, and compliance. Adverse effects were also evaluated. Review papers, case series, editorials, monographs, animal studies, in vitro studies, uncontrolled trials, letters to editors, case reports were omitted.

### 2.2. Database and Search Strategies

Three electronic databases were searched by independent reviewers: PubMed, Cochrane library and Scopus. Combinations of various keywords like “oral candidiasis” OR “oral candidosis” OR “oropharyngeal candidiasis” OR “candidal stomatitis” AND “nystatin” OR “antifungal agents” OR “polyenes” were used to extract all pertinent studies. Manual searches had also been conducted as a supplement.

### 2.3. Data Extraction and Quality Assessment

The scanning of titles and abstracts, selection of studies, reading full reports, data extraction and quality assessment was independently done by the two review authors (A.R., S.P.). All the pertinent data of each included study, including author, year of publication, region, study design, risk factors, characteristics of the patients like age and gender, detailed interventions, recall periods, outcomes, and adverse effects, were extracted and summarized in a table format using Excel Spreadsheet (Microsoft, Redmond, WA, USA) for qualitative synthesis.

The quality of the included studies was assessed using the Cochrane Handbook for Systematic Review of Interventions and the Rev Man 5.4.1 software. The following assessment criteria were used to assess the quality of the studies: (1) random sequence generation (if the study did not use this method, it was considered to have a selection bias); (2) allocation concealment (selection bias); (3) blinding of participants and personnel (performance bias); (4) blinding of outcome assessment (detection bias); (5) incomplete outcome data (attrition bias); and (6) selective reporting (reporting bias). The Kappa coefficient was used to calculate inter-rater agreement with regard to study inclusion and quality assessment. A third reviewer (S.R.M.) made an assessment when the two review authors could not reach a consensus. Each of the six points in every included study was assessed and colored ‘green’ for low risk, ‘yellow’ for unclear, and ‘red’ for high risk were appraised. The risk of bias was categorized as low when the study was showing more and equal to 60% of the ‘green’ score and high when there was 40% of either ‘yellow’ or ‘red’.

### 2.4. Data Synthesis and Analysis

The efficacy of nystatin versus PDT was evaluated using the Rev Man 5.4.1 software. Results were expressed as standard deviation (SD) together with the 95% confidence interval (CI), and plotted on a forest plot. I^2^ test was performed on the eligible studies and the value of the test <30% was considered low, 30%–70% was considered moderate and >70% was considered high heterogeneity of the effect size among the studies. The data extracted were from the various demographics; therefore, the random effect model was employed. A descriptive study was conducted on studies evaluating the efficacy of nystatin versus other antifungal treatments due to the limited number of studies or marked heterogeneity in many aspects of the study characteristics.

## 3. Results

### 3.1. Databases Search Results

**Figure 1:**
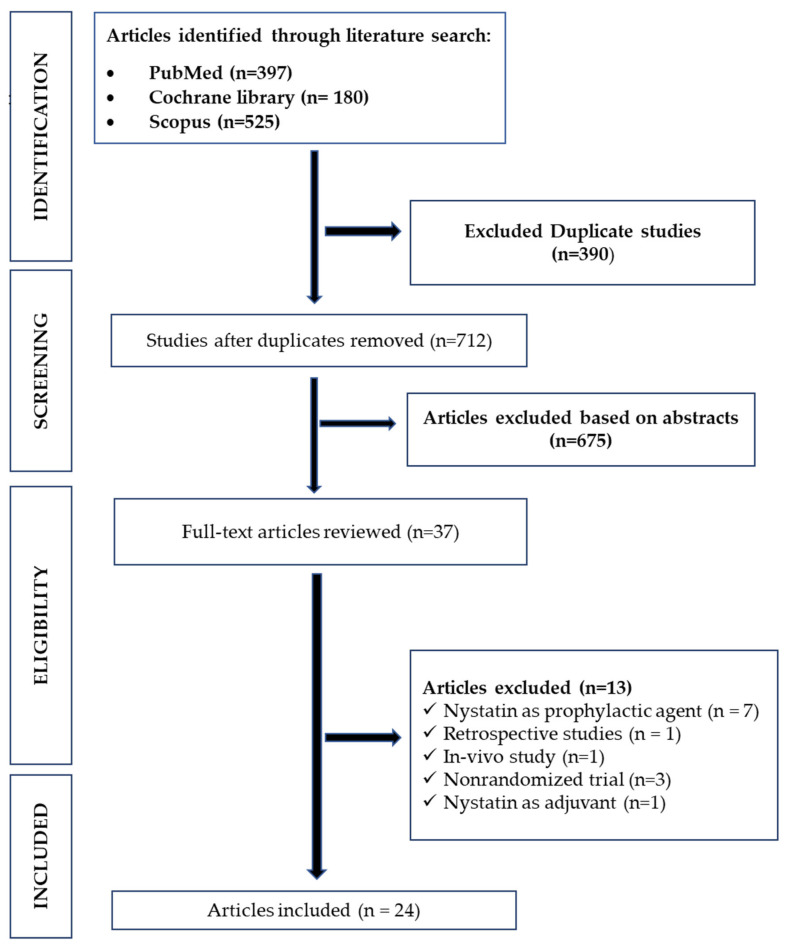
Study selection and PRISMA flowchart.

A total of 1102 abstracts were extracted from the three databases PubMed, Cochrane, Scopus (Figure 1).

After screening, three hundred ninety duplicate studies and six hundred seventy-five irrelevant studies were removed. On the basis of the eligibility criteria, thirteen studies were excluded: seven studies with nystatin as prophylactic agent, three nonclinical studies, one retrospective, one in vivo study, and one study with nystatin as adjuvant.

Finally, only 24 studies with a total of 1746 patients were included in the present analysis, with the average age of participants ranging from 12 months to 70 years.

### 3.2. Characteristics of the Included Studies

Fifteen trials were performed in patients with denture stomatitis [28,29,30,31,32,33,34,35,36,37,38,39,40], in which one was on patients with respiratory disorders [41] and another with diabetes mellitus [42]; four trials were conducted on infants or children [43,44,45,46], three trials included Human Immunodeficiency (HIV) or Acquired immune deficiency syndrome (AIDS) patients [47,48,49]; one trial was on hospitalized cancer patients and one trial were performed in multigroup patients (having diverse diseases like erythematous candidiasis, HIV, Xerostomia, organ transplantation and denture stomatitis) [50] (Table 1). These studies have compared nystatin with different interventions like Fluconazole, photodynamic therapy (PDT), microwave, placebo, etc., and they have been designated as controls. Six trials had compared nystatin with two different controls [36,37,38,41,46,48], while the rest had a single control. Nystatin was used in the suspension, gel, and pastille forms; the dosage ranged from 100,000 IU to 1,100,000 IU, three to five times a day, and the treatment duration was 10 to 30 days.

### 3.3. Risk of Bias and Quality of the Included Studies

**Figure 2:**
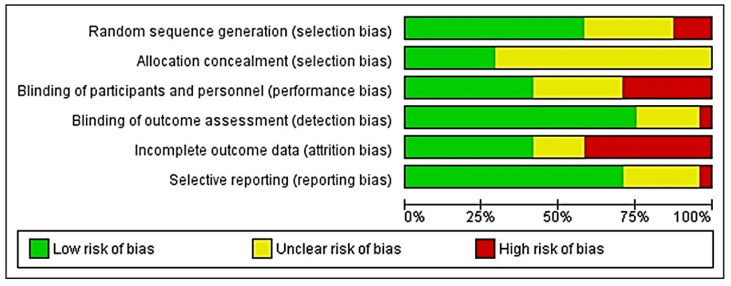
Depicting the plot of the distribution of review authors’ judgments across studies for each risk of bias item.

**Figure 3:**
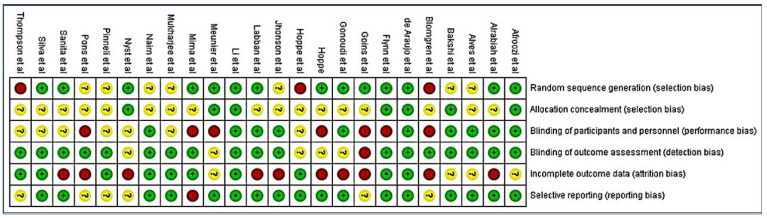
A summary table of review authors’ judgments for each risk of bias item for each study.

Only two of the included studies met all the seven assessment criteria [34,35]. Most studies were found to have an unclear risk of selection, performance and attrition bias, and a moderate risk of other biases. The overall risk of each bias is presented in Figure 2, and the risk of each bias in each of the studies separately is presented in Figure 3. A 100% agreement was achieved on study quality among the reviewers.

### 3.4. Potency Evaluation

The clinical and mycologic cure rates associated with both nystatin and the different controls have been summarized in Table 2.

**Figure 4:**
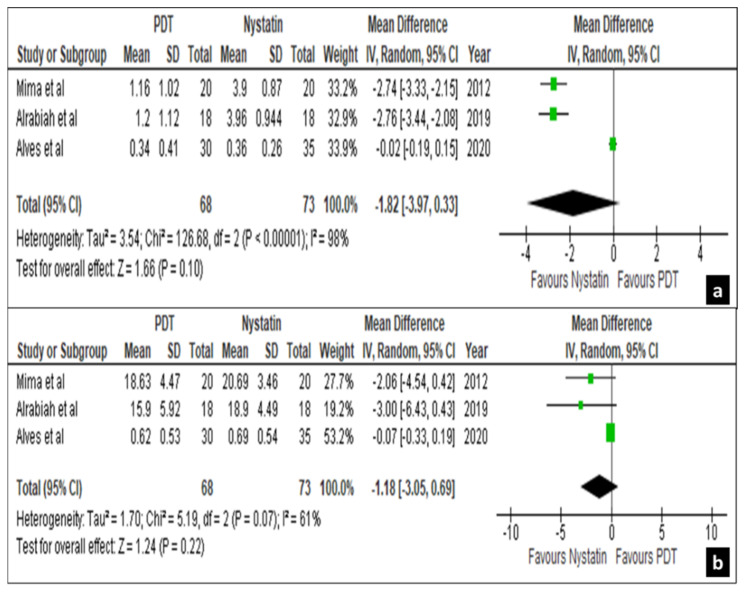
Forest plot comparing the efficacy of nystatin and PDT on palatal mucosa (**a**) and denture surface (**b**) in 15 days.

Only four out of the twenty-four trials comparing the efficacy of nystatin suspension with photodynamic therapy (PDT) were eligible for meta-analysis with mycological cure rates on the palatal mucosa and denture surfaces as an outcome [29,30,33,40]. Since, Labban et al. [33] used two forms of PDT (one used rose Bengal mediated and the other using curcumin), the studies were marked as Labban et al. A and B, respectively. Then, the results of mycological cure rates at different follow-up periods were expressed into proportions and mean depending on the data extracted from the individual studies. On day 15, three out of four studies, i.e., Mima et al. [40], Alrabiah et al. [29] and Alves et al. [30] showed a non-significant difference in the treatment effects on palatal mucosa and denture surfaces with a mean difference of −1.82 [−3.97, 0.33] and −1.18 [−3.05, 0.69], respectively, at a confidence interval of 95% (Figure 4a,b).

**Figure 5:**
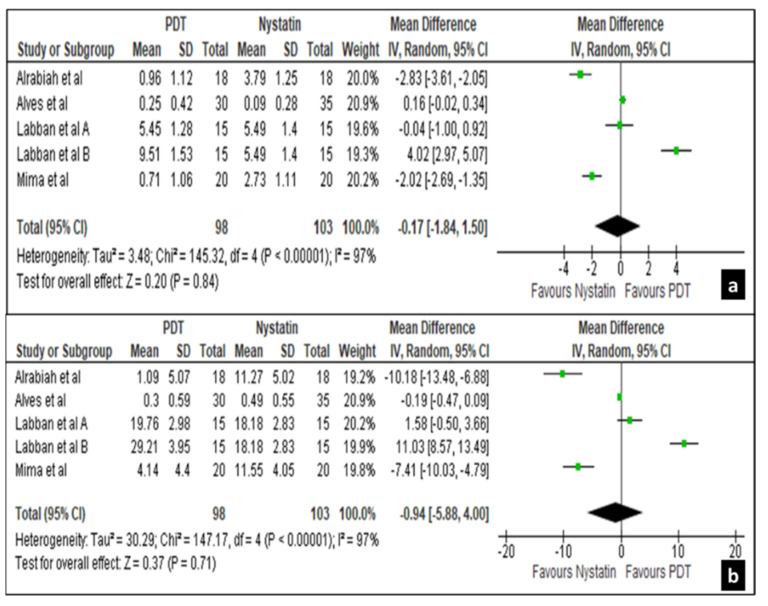
Forest plot comparing the efficacy of nystatin and PDT on palatal mucosa (**a**) and denture surface (**b**) in 30 to 45 days.

Between 30 to 45 days intervals all 4 studies revealed a non-significant difference in the treatment effects in mycological cure rates for both palatal mucosa and denture surfaces with a mean difference of −0.17 [−1.84, 1.50] and −0.94 [−5.88, 4.00], respectively, at 95% confidence interval (Figure 5a,b).

**Figure 6:**
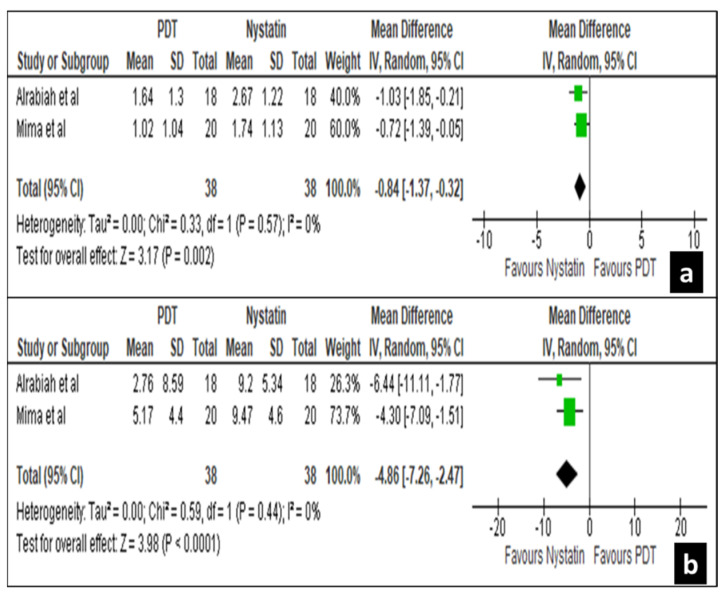
Forest plot comparing the efficacy of nystatin and PDT on palatal mucosa (**a**) and denture (**b**) surface in 60 days.

At 60 days interval two studies by Alrabiah et al. [29] and Mima et al. [40] revealed significant treatment effects for both palatal mucosa and denture surfaces with a mean difference of −0.84 [−1.37, −0.32] and −4.86 [−7.26, −2.47] at a confidence interval of 95, respectively (Figure 6a,b).

**Figure 7:**
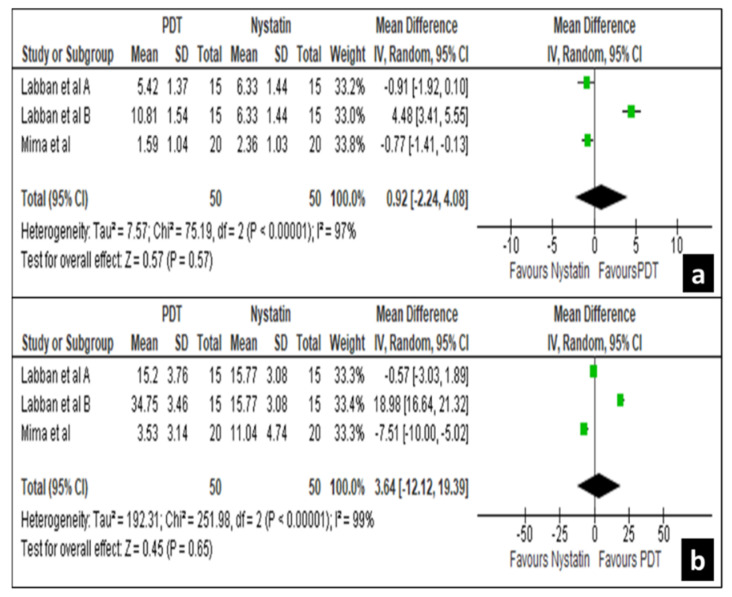
Forest plot comparing the efficacy of nystatin and PDT on palatal mucosa (**a**) and denture (**b**) surface in 80 to 95 days.

Again between 80 to 90 days intervals, two studies, i.e., Labban et al. [33] and Mima et al. [40] revealed a non-significant difference in the treatment effects for both palatal mucosa and denture surfaces with a mean difference of −0.92 [−2.24, 4.08] and 3.64 [−12.12, 19.39] at a confidence interval of 95% (Figure 7a,b). These results show that nystatin suspension has similar clinical efficacy as that of photodynamic therapy.

Due to marked heterogeneity in many aspects of the study characteristics in all other included studies meta-analysis was not conducted. The efficacy of nystatin suspension was compared to other antifungal agents in five trials [43,44,46,47,48]. The results showed that the clinical, as well as mycological efficacy of nystatin suspension was 9%–67.8% and 5.6%–13%, respectively, whereas the clinical and mycological efficacy was higher with fluconazole (87%–100% and 60%–76%, respectively) [43,44,49,50], miconazole (99% and 54.1%, respectively) [45], and ketoconazole (43% and 57%, respectively) [51] in infants and children for the treatment of oral candidiasis. The clinical and mycological efficacy was higher with miconazole (99% and 54.1%, respectively) [46], and gentian violet (42%–68.5% and 62%, respectively) [47] in HIV/AIDS patients with oral candidiasis. Two studies compared the efficacy of nystatin pastilles to placebo and observed 14.3%–76.9% of clinical cure rates and 40%–71.4% of mycological cure rates [37,39]. One of the trials compared the efficacy of nystatin pastilles and amphotericin B and the results showed that both have similar clinical efficacy of 76.9% and 88.8%, respectively [37]. Another study [39] demonstrated a comparatively higher clinical cure rate for nystatin pastilles (87%) than that of nystatin suspension (80%). Two studies [36,42] compared the clinical efficacy of nystatin suspension to denture irradiation and the results showed that nystatin suspension had a clinical cure rate of 18.75%–20%, whereas the clinical cure rates of denture irradiation were 22.22%–25%. The results of the above trials point towards the higher clinical and mycological efficacy of fluconazole, miconazole, gentian violet, and ketoconazole as compared to that of nystatin.

### 3.5. Duration, Dosage, Formulations and Adverse Effects

There was a lot of diversity in the study designs and the treatment protocols (dose, duration of treatment, formulation used) of the included trials. Hence, only four trials qualified for meta-analysis, for the rest only descriptive study was performed. Nystatin was used in pastille, suspension, solution, gel and a combination of pastille and suspension forms in the twenty-four studies. Amongst the denture stomatitis patients, the clinical cure rate with solution form was 89%, with suspension form was 54% and with pastille was 28%. On increasing the dosage from 200,000–400,000 IU to 500,000 IU the clinical cure rate increased up to 77% while using pastilles. The mycologic cure rate with the paste form was 30.7%. The clinical cure rate was high on using pastilles form in respiratory patients at 87% and 87.5% in cancer patients when using both pastille and suspension forms. Meunier et al. [51] observed that the clinical and mycologic cure rates were 87.5% and 66% of cancer patients, respectively, with oral *Candida* infection on using a combination of nystatin pastilles and suspension. Trials conducted on infants, children, HIV and AIDS patients with oral candidiasis exhibited a clinical cure rate of 9%–63.5% and the mycologic cure rate was 6–13% with the use of suspension form. It was observed that pastilles worked better than suspension or solution (Table 3).

Six trials [31,37,43,45,49,50] out of twenty-four reported adverse effects after the administration of nystatin, while the remaining eighteen trials did not mention any adverse effects as secondary outcomes. Nausea, vomiting, unpleasant taste and diarrhea were the most common adverse effects in both nystatin as well as the control group (Table 4).

## 4. Discussion

Oral candidiasis happens to be the most prevalent opportunistic infection in the oral cavity, often known as the “disease of the diseased” [6,52]. It commonly affects infants and aged individuals, especially denture wearers [53]. However, it can affect individuals of any age having local or systemic factors which predispose them to *Candida* infection [54]. Often uncontrolled diabetes mellitus, xerostomia due to different systemic causes, long-term glucocorticoid therapy, immunodeficiency states, patients with hematologic disorders or patients with oral malignancies on radiotherapy or chemotherapy develop oral candidiasis [55]. Even local factors like poor oral hygiene, oral sepsis or any mucosal alterations like ulceration/growth and tissue abuse habits like smoking can predispose to oral candidiasis [20]. It is imperative to manage candidiasis of the oral cavity on time before it spreads to contiguous mucosa like the upper respiratory tract, esophagus, blood, or even spread to the central nervous system [12,17]. It can also cause systemic infection which may be life-threatening. Besides the *Candida,* metabolites can lead to Id reaction which is a type of hypersensitivity reaction leading to eczema, bronchial asthma, and gastric irritation [3].

Since the incidence and the prevalence of oral candidiasis have been on the rise in recent years, the use of antifungals has been on the rise [6]. Both topical and systemic antifungal therapies for the patients have been advocated depending on the severity of the disease, though topical therapies are preferred over systemic ones due to the renal and hepatotoxicity associated with the latter [20,21]. Even with the availability of newer antifungal drugs, polyenes like itraconazole, voriconazole, posaconazole isavuconazole and echinocandins like caspofungin, micafungin, anidulafungin which have lesser side effects and more clinical options, topical antifungal therapy using nystatin is the treatment of choice owing to its efficacy, cost and less adverse effects [14].

Nystatin, a polyene antibiotic, interacts with the ergosterol in the fungal cell membrane making it porous and vulnerable to lysis, thus exerting its antifungal effect [56].

The clinical practice guidelines for the management of oral candidiasis were given by the Infectious Diseases Society of America recommending the usage of nystatin suspension having a concentration of 100,000 IU/mL with a dosage of 4–6 mL four times a day or one to two nystatin pastilles (200,000 IU) to be given four times a day for one to two weeks for mild oral/oropharyngeal candidiasis [57]. World Health Organization (WHO) has recommended topical therapies having nystatin either in the form of suspension or pastilles are good substitutes to orally administered fluconazole for treating oral and oropharyngeal candidiasis even in HIV-positive individuals [58]. However, the usage of nystatin, its availability and its administration vary in different countries and populations.

The duration of the antifungal treatment plays a vital role in its efficacy. Short term therapies of nystatin did not yield good results in case of infants or HIV/AIDS patients. Nystatin administered at a concentration of 0.25 to 1 times the minimum inhibitory concentration value for half an hour results has been reported to have better antifungal effect up to 6 h in *Candida* isolates [59]. Since *Candida* species adhere to the oral mucosal cells and colonize, topical antifungals like nystatin getting absorbed into the oral epithelium are more effective as compared to oral antifungals. Like in previous studies, a treatment duration of 4 weeks was considered clinically more effective in our descriptive analysis.

The present systematic review points towards the efficacy of nystatin being comparable to different other topical therapies in decreasing the mycological loads of *Candida* on the palatal and denture surfaces in oral candidiasis. Because of the heterogenicity of the data present in various studies, meta-analysis was possible for only four studies. The clinical efficacy of nystatin was significantly increased over PDT at a treatment duration of 60 days on both the palatal mucosa and denture surfaces while it was at par during treatment intervals of 15, 30 to 45 and 80 to 90 days. Insignificant results were shown by only one of the studies, i.e., Alves et al. [30] which was included in the meta-analysis. The higher heterogeneity observed could be attributed to the fact that the study by Alves et al. [30] had a larger sample size and did not use a secondary culture medium, i.e., sabouraud dextrose agar unlike the others. Nystatin and photodynamic therapy are equally effective for the clinical remission of denture stomatitis as well as in significant reduction of CFU/mL of *Candida* species from dentures and palatal surfaces of the patients. Additionally, both the therapies have equal effects against *Candida albicans*, which had the highest prevalence among all species in denture stomatitis. The principal advantage of photodynamic therapy is that, unlike antifungal agents, development of resistance to photodynamic therapy seems unlikely due to its mechanism of action and has no toxic adverse effects. In immunocompromised states like HIV/AIDS where the continuous use of antifungal regimens has led to drug resistance and poor patient tolerability, PDT could be effective as it is not toxic to the host cells and be used for recurrent oral candidiasis [33].

A similar systematic review was conducted in 2016 [25] assessing the efficacy of different antifungal agents taking into account the various formulations, dosages and duration for treating oral candidiasis. They had searched four databases and ultimately included only eleven trials for the qualitative analysis. A meta-analysis was conducted on two studies comparing the efficacy of nystatin pastilles to placebo in the treatment of denture stomatitis which showed nystatin pastilles were superior to placebo. Another meta-analysis included three trials which compared the efficacy of nystatin suspension with fluconazole in treating oral/oropharyngeal candidiasis in infants/children found fluconazole was significantly superior to nystatin. Finally, descriptive analysis was conducted on seven studies which were not included in meta-analysis due to limited sample size or marked heterogeneity. Their results indicated that nystatin suspension was at par in clinical efficacy when compared to interventions like PDT and sodium benzoate but inferior to miconazole, ketoconazole and gentian violet in infants, children and HIV/AIDS patients. Nystatin pastilles were also similar in efficacy in comparison with amphotericin B and ketoconazole.

Like the present study, even in the systematic review conducted by Lyu et al. [25], it was found that nystatin was superior to placebo in treating denture stomatitis, but in infants and immunocompromised patients fluconazole was more effective than nystatin suspension for oral candidiasis. They also concluded that a combination of nystatin pastilles and suspension for two weeks had a better clinical and mycologic cure rate compared to the usage of nystatin suspension alone. The common adverse effects reported by them were also bitter taste and gastrointestinal upset akin to the present study.

There are several limitations to this meta-analysis. First, very few clinical trials with homogeneity were available. Second, several studies were considered to be at a high risk of selection, performance and attrition bias, and a moderate risk of other biases. The majority of the studies did not provide enough information about allocation concealment. The inconsistent quality of the included studies would impact the credibility of the results. These deficiencies indicate that well-designed and high-quality randomized controlled trial studies are needed in the future.

## 5. Conclusions

Antifungal therapies have evolved over time. Nowadays, newer triazoles and echinocandins have high range of antifungal activity with better patient acceptance in terms of ease of dosage and better tolerability, especially in recurrent/recalcitrant cases. Yet, topical therapy using nystatin is still the mainstay for the treatment of oral candidiasis, because of its increased efficacy, low cost, and less side effects. Photodynamic therapy is an expensive option, though it is quite efficacious. Therefore, nystatin suspensions can be the treatment regimen of choice for denture stomatitis, as the present descriptive analysis highlights the equal efficacy of 100,000 IU of nystatin suspension and six sessions of PDT.

## Figures and Tables

**Table 1:** Characteristics of the included studies.

Author/Country	Risk Factor	Nystatin Group			Control Group			Nystatin Group				Control Group				
		Age (Mean)	Sex	N	Age (Mean)	Sex	N	Formulation	Dose	Frequency (Times/day)	Duration (Days)	Medication	Formulation	Dose	Frequency	Duration (Days)
Afroozi et al. 2019 [28], Iran	Denture	67.4 y	-	33	67.6 y	-	33	Sol	100,000 IU	3 times a day	15	PDT	-	-	2 sessions	15
Alrabiah et al. 2019 [29], Saudi Arabia	Denture	-	-	18	-	-	18	Susp	100,000 IU	4 times a day	14	PDT	-	-	twice in one week	30
Alves et al. 2020 [30], Brazil	Denture	69 y	-	35	70 y	F = 19 M = 11	30	Susp	100,000 IU/mL)	4 times a day	15	PDT	-	-	6 sessions	15
Araújo et al. 2021 [35], Brazil	Denture	57 y	-	18	-	-	18	Sol	1,000,000 IU	3 times a day	15	CZ	oral spray	-	3 times a day	15
Bakhshi et al. 2012 [31], Iran	Denture	73.52 y	-	20	-	-	20	Sol	100,000 IU/ml	3 times a day	30	GE	Sol	40 mg/ml	3 times a day	30
Gonoudi et al. 2021 [32], Iran	Denture	60.93 y	-	14	55.86 y	-	14	Susp	100,000 IU	4 times a day	14	ZM	Sol	5 ml	5 times a day	14
Johnson et al. 1989 [39], USA	Denture	-	-	8	-	-	8	Pas	200,000 IU	5	14	Placebo	Pastilles		5	14
		-	-	8	-	-	8	Pas	400,000 IU	5	14	Placebo	Pas		5	14
Labban et al. 2021 [33], Saudi Arabia	Denture	56.9 y	-	15	57.2 y	-	15	Susp	100,000 IU/mL	4 times a day	15	PDT	-	-	6 sessions	15
Li et al. 2014 [34], China	Denture	64.84 y	F = 24 M = 7	31	62.72 y	F = 29 M = 5	34	Paste	2%	3	30	NYT + Pb	Paste + Lozenges		3	30
Mima et al. 2012 [40], Brazil	Denture	62.45 y	-	20	61.25 y	-	20	Susp	100 000 IU	4 times a day	15	PDT	-	-	6 sessions	15
Nairn et al. 1975 [37], England	Denture	-	-	13	-	-	18	Pas	500,000 IU	4	30	AMB	Lozenges	10 mg	4	30
		-	-	13	-	-	15	Pas	500,000 IU	4	30	Placebo				
Pinelli et al. 2013 [38], Brazil	Denture	81.4 y	-	10	-	-	10	Sol	100,000 IU	4 times a day	30	RC	Sol	-	-	30
		81.4 y	-	10	-	-	10	Sol	100,000 IU	4 times a day	30	MIC	Gel		4 times a day	30
Silva et al. 2012 [36], Brazil	Denture	62.5 y	-	20	59.5 y	-	20	Susp	100,000 IU/ml	4	14	DM	Irr		Once per week	14
		62.5 y	-	20	56.8 y	-	20	Susp	100,000 IU/ml	4	14	DM	Irr		3 times per week	14
Sanita et al. 2012 [42], Brazil	Denture in diabetic patients	62.6 y	-	10	62.2 y	-	10	Susp	100,000 IU/m	4	14	DM	Irr		3 times per week	14
Thompson et al. 1986 [41], England	Respiratory disease	59 y	-	18	-	-	18	Pas	100,000 IU	4	7	NYT	Susp	100 000 units	4	7
Goins et al. 2002 [44], USA	Infants	1–12 mon	-	28	1–12 mon	-	17	Susp	100,000 IU	4	10	FLC	Susp		1 per day	7
Hoppe 1997 [46], Multicenter study	Infants	130 days	F = 0 M = 77	85	132 days	F = 0 M = 95	98	Susp	100,000 IU	4	12	MIC	Gel		4	12
Hoppe et al. 1996 [45], Multicenter study	Infants	5 months	-	35	5 mon	-	27	Gel	250,000 IU	4	14	MIC	Gel		4	14
		5 months	-	35	5 mon	-	27	Gel	100,000 IU	4	14	MIC	Gel		4	14
Flynn et al. 1995 [43], USA	Infants Children	6 months–13 y	-	88	6 mon–13 y	-	94	Susp	400,000 IU	4	14	FLC	Susp			14
Meunier et al. 1990 [51], Belgium	Cancer patients	-	F = 10 M = 14	24	-	F = 8 M = 10	18	Susp + Pas	1000,000 IU + 100,000 IU	3	10 to 12	KCZ	Tab			10 to 12
Mukherjee et al. 2017 [47], Multicenter study	HIV	-	F = 66 M = 45	111	-	F = 62 M = 48	110	Susp	500,000 IU	4	14	GV	Sol			14
Pons et al. 1997 [49], USA	HIV, AIDS	38 y	-	84	38 y	-	83	Susp	500,000 IU	4	14	FLC	Susp			14
Nyst et al. 1992 [48], Zaire	AIDS	35.4 y	-	47	34.5 y	-	49	Susp	200,000 IU	4	14	GV	Susp			14
		35.4 y	-	47	34.5 y	-	45	Susp	200,000 IU	4	14	KCZ	Troche			14
Blomgren et al. 1998 [50], Sweden	Multigroup patients	60.7 y	-	33	58.4 y	-	34	Sol	100,000 IU	4	21	FLC	Cap			7

HIV = human immunodeficiency virus; AIDS = acquired immunodeficiency syndrome; PDT—Photodynamic therapy; CZ—Cinnamomum zeylanicum; GE—Garlic extract; ZM—Zataria multiflora; NYT—nystatin; Pb—probiotics; AMB—Amphotericin B; RC—Ricinus communis; MIC—Miconazole; DM—Denture microwave; FLC—Fluconazole; KCZ—Ketoconazole; GV—Gentain violet; Susp—Suspension; Sol—Solution; Tab—Tablet; Cap—Capsule; Irr—Irradiation; Pas—Pastilles.

**Table 2:** Clinical and mycologic potency of nystatin and the control treatments.

Authors	Risk Factors	Clinical Cure Rates		Mycological Cure Rates	
		Nystatin	Controls	Nystatin	Controls
Afroozi et al. 2019 [28]	Denture	89.30%	53.60%	-	-
Alrabiah et al. 2019 [29]	Denture	-	-	-	-
Alves et al. 2020 [30]	Denture	54.20%	53.30%	-	-
Bakhshi et al. 2012 [31]	Denture	-	-	-	-
Araújo et al. 2021 [35]	Denture	89%	61%	83%	33%
Gonoudi et al. 2021 [32]	Denture	-	-	-	-
Johnson et al. 1989 [39]	Denture	28.60%	0	57.10%	0
		14.30%	0	71.40%	0
Labban et al. 2021 [33]	Denture	-	-	-	-
Li et al. 2014 [34]	Denture	-	-	30.77%	20%
Mima et al. 2012 [40]	Denture	53%	45%	-	-
Nairn et al. 1975 [37]	Denture	76.90%	88.80%	40%	6.25%
		76.90%	40%	40%	20%
Pinelli et al. 2013 [38]	Denture	-	-	-	-
Silva et al. 2012 [36]	Denture	18.75%	23.53%	-	-
		18.75%	22.22%	-	-
Sanita et al. 2012 [42]	Denture in diabetic patients	20%	25%	-	-
Thompson et al. 1986 [41]	Respiratory disease and dentures	87%	80%	-	-
Goins et al. 2002 [44]	Infants	28.60%	100%	5.60%	73.30%
Hoppe et al. 1996 [45]	Infants	42.80%	85.10%	20%	29.60%
		48.50%	85.10%	3.00%	29.60%
Hoppe 1997 [46]	Infants	54.10%	99%	8.20%	54.10%
Flynn et al. 1995 [43]	Infants, Children	51%	91%	11%	76%
Meunier et al. 1990 [51]	Cancer patients	72%	87%	24%	61%
Mukherjee et al. 2017 [47]	HIV	67.80%	68.50%	-	-
Pons et al. 1997 [49]	HIV, AIDS	52%	87%	6%	60%
Nyst et al. 1992 [48]	AIDS	9%	42%	13%	62%
		9%	43%	13%	57%
Blomgren et al. 1998 [50]	Multigroup patients	16.70%	30%	-	-

**Table 3:** Summary of the use and potency of nystatin.

Risk Factor	Formulation	Dose	Frequency (Times/Day)	Duration (Days)	Clinical Cure Rates (%)	Mycological Cure Rates (%)
Denture	Susp	100,000 IU	4	15	54.2	
Denture	Sol	100,000 IU	3	15–30	89.3	
Denture	Pas	200,000–400,000 IU	5	14	28.6–14.3	57.10–71.4
Denture	Pas	500,000 IU	4	30	76.9	40
Denture	Paste	2%	3	30		30.77
Denture in diabetic patients	Susp	100,000 IU	4	14	20	-
Respiratory disease	Pas	100,000 IU	4	7	87	-
Infants and children	Susp	100,000–400,000 IU	4	10 to 14	28.6–54.1	5.6 – 11
Infants and children	Gel	250,000 IU	4	14 days	42.8/48.5	20/3.0
Cancer	Susp + Pas	1,000,000 IU + 100,000 IU	3	10 to 12	87.5	66
HIV/AIDS	Susp	100,000–500,000 IU	4	14	9–67.8	6–13
Multigroup	Susp	100,000 IU	4	21	16.7	-

Susp—Suspension; Sol—Solution; Pas—Pastille.

**Table 4:** Adverse effects of nystatin and controls.

Author	Risk Factor	Adverse Effects in Nystatin Group	Adverse Effects in Control Group
Bakhshi et al. 2012 [31]	Denture	nausea in 6, vomiting in 1, diarrhea in 5, anorexia in 1, burning in 1	itching in 1
Nairn et al. 1975 [37]	Denture	unpleasant taste in eight patients	unpleasant taste in five patients
Hoppe et al. 1996 [45]	Infants	vomiting in one patient	vomiting in two patients
Flynn et al. 1995 [43]	Infants, Children	three patients (vomiting, nausea, diarrhea, anorexia, abdominal pain), one patient (rash, headache)	six patients (vomiting, nausea, diarrhea, anorexia, abdominal pain), one patient (rash, headache)
Pons et al. 1997 [49]	HIV, AIDS	nausea, vomiting and diarrhea	nausea in one patient, and elevated liver enzyme concentrations in two patients
Nyst et al. 1992 [48]	AIDS	-	irritation and small superficial oral ulcers in two patients
Blomgren et al. 1998 [50]	Multigroup patients	nausea in one patient	-

HIV = human immunodeficiency virus; AIDS = acquired immunodeficiency syndrome.

## Data Availability

Not applicable.
